# The impact of nutritional counseling on thyroid disorders in head and neck cancer patients after (chemo)radiotherapy: results from a prospective interventional trial

**DOI:** 10.1007/s00066-021-01865-3

**Published:** 2021-11-01

**Authors:** Anastassia Löser, Kerstin Ramke, Maximilian Grohmann, Linda Krause, Pia Roser, Franziska Greinert, Anna Finger, Margaret Sommer, Eva Culmann, Tessa Lorenz, Saskia Becker, Marvin Henze, Daniel Schodrok, Julia von Grundherr, Silke Tribius, Andreas Krüll, Cordula Petersen

**Affiliations:** 1 Outpatient Center of the UKE GmbH, Department of Radiotherapy and Radiation Oncology, University Medical Center Hamburg-Eppendorf, Martinistraße 52, 20246 Hamburg, Germany; 2 Institute of Medical Biometry and Epidemiology, University Medical Center Hamburg-Eppendorf, Martinistraße 52, 20246 Hamburg, Germany; 3 Center for Internal Medicine, Department of Nephrology, Rheumatology and Endocrinology, University Medical Center Hamburg-Eppendorf, Martinistraße 52, 20246 Hamburg, Germany; 4 University Medical Center Hamburg-Eppendorf, University Cancer Center Hamburg (UCCH), Martinistraße 52, 20246 Hamburg, Germany; 5 Hermann Holthusen Institute for Radiation Oncology, Asklepios Hospital St. Georg, Lohmühlenstraße 5, 20099 Hamburg, Germany; 6 Department of Radiotherapy and Radiation Oncology, University Medical Center Hamburg-Eppendorf, Martinistraße 52, 20246 Hamburg, Germany

**Keywords:** Thyroid gland, Normal tissue complication probability (NTCP) model, Chemoradiotherapy, Radiotherapy, Hypothyroidism

## Abstract

**Objective:**

To analyze the impact of nutritional counseling on the development of hypothyroidism after (chemo)radiotherapy in head and neck cancer patients to propose a new normal tissue complication probability (NTCP) model.

**Materials and methods:**

At baseline, at the end of (chemo)radiotherapy, and during follow-up, thyroid-stimulating hormone (TSH) with free thyroxin (fT3 and fT4), nutritional status, and nutrient intake were prospectively analyzed in 46 out of 220 screened patients. Patients received (chemo)radiotherapy within an intervention (individual nutritional counseling every 2 weeks during therapy) and a control group (no nutritional counseling).

**Results:**

Overall median follow-up was 16.5 [IQR: 12; 22] months. Fourteen patients (30.4%) presented with hypothyroidism after 13.5 [8.8; 17] months. During (chemo)radiotherapy, nutritional status worsened in the entire cohort: body mass index (*p* < 0.001) and fat-free mass index (*p* < 0.001) decreased, calorie deficit (*p* = 0.02) increased, and the baseline protein intake dropped (*p* = 0.028). The baseline selenium intake (*p* = 0.002) increased until the end of therapy. Application of the NTCP models by Rønjom, Cella, and Boomsma et al. resulted in good performance of all three models, with an AUC ranging from 0.76 to 0.78. Our newly developed NTCP model was based on baseline TSH and baseline ferritin. Model performance was good, receiving an AUC of 0.76 (95% CI: 0.61–0.87), with a sensitivity of 57.1% and specificity of 96.9% calculated for a Youden index of 0.73 (*p* = 0.004; area = 0.5).

**Conclusion:**

Baseline TSH and ferritin act as independent predictors for radiotherapy-associated hypothyroidism. The exclusion of such laboratory chemistry parameters in future NTCP models may result in poor model performance.

## Introduction

Although radiotherapy constitutes a curative therapeutic approach for squamous cell carcinoma of the head and neck (HNSCC), it is often associated with a significant dose to the thyroid gland. Radiotherapy-induced thyroid dysfunction can lead to primary manifest hypothyroidism (low free thyroxin [fT4] and elevated thyroid-stimulating hormone [TSH]) or subclinical (normal fT4 and elevated TSH) hypothyroidism, Hashimoto’s thyroiditis, or Graves’ disease. Primary hypothyroidism is considered the most common radiotherapy-induced thyroid disorder and affects 20–60% of patients receiving irradiation to the neck [1, 2]. It usually occurs within 5 years after therapy completion [1]. Prior thyroidectomy and the applied irradiation dose are risk factors for hypothyroidism [1, 3, 4]. A mean thyroid dose (D_mean_) of ≤ 30 Gy is considered an important threshold dose for maintaining thyroid function [5]. Also, a V45Gy of less than 50% and more than 50% is associated with a 1-year incidence of hypothyroidism of 22.8% and 56.1%, respectively [6]. Apart from the applied radiation dose, the occurrence of hypothyroidism correlates with a reduction in thyroid volume [2, 7, 8].

In addition to dosimetric factors, deficiency symptoms of certain macro- and micronutrients may occur in the context of tumor- or treatment-related malnutrition, favoring the development of hypothyroidism [9]: Among these are iodine (important component of fT3 and fT4), iron (required for thyroid hormone formation) [9–13], selenium (regulation of the conversion of fT4 to fT3) [14], and amino acids (needed for the formation of free thyroid hormones) [15].

Between 3 and 52% of patients with HNSCC are malnourished before therapy initiation [16–18]. Treatment-related toxicity often leads to further progression of malnutrition to as much as 88% [16, 17]. Apart from nutritional screening tools and anthropometric methods (e.g., BMI, calf circumference), bioelectrical impedance analysis (BIA) is becoming increasingly established to assess nutritional status and measure body composition.

Data on thyroid disorders in head and neck cancer patients undergoing (chemo)radiotherapy receiving nutritional intervention are completely lacking. The aim of this prospective intervention study was to identify possible predictors of hypothyroidism by considering dosimetric as well as clinical and nutritional factors to propose a new multivariable normal tissue complication probability (NTCP) model to predict radiation-induced hypothyroidism [7, 19].

## Methods

### Study design

The data analyzed for this study were prospectively obtained during a monocentric, controlled randomized (1:1) intervention study performed at the University Medical Center Hamburg-Eppendorf. This prospective trial was named “HEADNUT-trial” standing for *head* and neck cancer patients undergoing *nut*ritional intervention [20]. This work focuses on radiotherapy-induced hypothyroidism and the impact of nutritional indicators to develop a new multivariable predictive model.

Patient recruitment was initiated in October 2018. Due to the upcoming corona pandemic, recruitment was paused between March and August 2020, and was later continued until October 2020. This study was approved by the local ethics committee (PV5818) and registered within the German Clinical Trials Register (DRKS00016862). All patients signed written informed consent.

This study consists of a control group and an intervention group. Patients within the intervention group received personalized nutritional counseling during (chemo)radiotherapy.

### Patient recruitment

Eligible patients presented with squamous cell carcinoma of the oropharynx, oral cavity, hypopharynx, larynx, or salivary glands, showing an euthyroid metabolic status at baseline. Exclusion criteria included another solid tumor within the last 15 years, palliative intent, a Karnofsky performance status below 60%, and pacemakers (as a relative contraindication for BIA).

### Laboratory analyses

The following laboratory parameters were measured in all patients at the beginning and the end of therapy: hemoglobin (normal range: 12.3–15.3 g/dL), TSH (normal range: 1.56–4.78 mU/L), fT4 (normal range: 11.5–22.7 pmol/L), fT3 (normal range: 3.5–6.5 pmol/L), ferritin level (normal range: 10–291 µg/L), and total protein (normal range: 57–82 g/L). During follow-up, TSH levels were prospectively collected at least once per year.

### Assessment of nutritional status

In both treatment arms, nutritional status was documented at the beginning and at the end of therapy. At both timepoints, all patients were clinically examined by a radiation oncologist and a dietician specialized in oncology assessed the individual nutritional risk profile (including anthropometric data with height and weight). Additionally, BIA (Biacorpus RX4004M; MEDI CAL HealthCare GmbH, Karlsruhe, Germany) was performed in all patients [21] and a 3-day food diary was submitted. Nutrient and calorie intake was interpreted by DGExpert software (v1.3.14.1, German Society for Nutrition [DGE], Bonn, Germany).

Only patients of the intervention group received nutritional consultations (lasting for 30 min) every 2 weeks with personalized nutritional recommendations based on their anthropometric measurements including BIA, their submitted food diaries, their clinical condition, the severity of potential therapy-associated side effects, and results from laboratory analyses.

Malnutrition was defined at a BMI of < 18.5 kg/m^2^ and a BIA-derived fat-free mass (FFM) index (FFMI = FFM/height^2^) of < 15 and < 17 kg/m^2^ in women and men, respectively [22]. Also, the phase angle was obtained from BIA [23, 24].

### Treatment planning, dosimetry, and (chemo)radiotherapy

A contrast-enhanced planning CT (Somatom, Siemens Healthcare, Forchheim, Germany) was obtained at 3‑mm slice thickness for each patient in supine position. Target volume and normal tissue contouring was performed on the planning CT using Eclipse (v15.1, Varian Medical Systems, Inc., Palo Alto, CA, US). During treatment planning, no dose restrictions to the thyroid gland were defined. For dosimetric evaluation, a corresponding dose–volume histogram (DVH), the thyroid volume, the mean (D_mean_), the minimum dose (D_min_), and the maximum thyroid dose (D_max_) were recorded. Also, V_10_, V_20_, V_30_, V_40_, V_45_, V_50_, V_60_, and V_70_ were calculated [6, 7]. CV_x_ (cm^3^) is defined as the thyroid volume (cm^3^) that received ≤ x Gy. We determined CV_10_, CV_20_, CV_30_, and CV_40_ [5, 25]_._

The thyroid gland was contoured on the planning CT and on all available pretherapeutic MRIs and on all follow-up CT and MRI scans using Eclipse. For the subsequent pre- versus posttherapeutic comparison of thyroid volumes, intermodal comparison was excluded, so only MRI scans were compared with MRI scans and CT scans with CT scans. To rule out interobserver variability, all thyroid glands were contoured by the same physician.

Radiotherapy was prescribed as intensity-modulated radiotherapy (IMRT) in single fractions of 1.7 to 2.0 Gy, five times per week, to cumulative doses from 60 to 70.4 Gy [26, 27]. For concomitant chemotherapy, either 3‑weekly cycles of cisplatin 100 mg/m^2^, weekly cisplatin 40 mg/m^2^, or a regimen with concomitant 5‑fluorouracil (5-FU; 600 mg/m^2^ on days 1 to 5) and mitomycin C (10 mg/m^2^ on days 5 and 36) was followed.

### Clinical and radiological follow-up

The first radio-oncological exam and the first follow-up MRI or CT (in case of contraindications for MRI) of the head and neck area were performed 6–8 weeks after therapy completion. A BIA measurement was also taken at this time. MRI/CT imaging of the head and neck area was repeated once per year along with a CT scan of the chest and abdomen. Radio-oncological follow-up examinations occurred semiannually.

### Statistical analyses

Our endpoint was defined as the occurrence of hypothyroidism (elevated TSH value: > 4.78 mU/L) at any time during follow-up. We did not differentiate between manifest and subclinical hypothyroidism. Follow-up started from the date of initial diagnosis of HNSCC.

The difference between the actual calorie intake and the calculated required calorie intake needed to maintain body weight (kcal) was defined as “calorie deficit” (∆ calorie deficit). The difference between a measured baseline value and the same value at the end of therapy was referred to as “∆” in each case (e.g., ∆ iodine intake = baseline iodine intake - iodine intake at therapy completion).

Normally distributed values were expressed as means with standard deviations (±SD), and nonnormally distributed variables were expressed as medians (interquartile range [IQR] with first and third quartiles). To evaluate differences within a 2 × 2 contingency table, Fisher’s exact test was chosen. In case of cross-tabulations with more variables, chi-square test was used. To test for differences between two independent samples, Mann–Whitney U test was applied. To compare the association between mean ranks, Wilcoxon-signed rank test was used.

The following logistic regression-based model has been described previously by several authors [7, 28, 29]:

$$NTCP=\left(1+e^{-S}\right)^{-1},\text{in which}$$

$$S=\beta _{0}+\beta _{1}\cdot x_{1}+\ldots +\beta _{n}\cdot x_{n}$$

*β*_0_ is the constant from the multivariable logistic regression model, while *β*_*n*_ is the regression coefficient multiplied with the corresponding input variable *x*_*n*_.

To estimate whether this logistic regression-based model is also suitable for the present dataset, we applied the NTCP models by Rønjom, Cella, and Boomsma et al. using MedCalc (version 19.6, MedCalc Software Ltd, Ostend, Belgium) [7, 28, 29]. Univariable logistic regression analyses were performed with SPSS (version 25.0, IBM Corp., Armonk, NY, US) and all variables shown in Table 2 were tested individually for differences in the endpoint. We established four separate multivariable regression models. In all four models, all variables from our univariable regression analyses with a *p* < 0.05 (baseline iodine intake, ∆ iodine intake, baseline TSH and ferritin, as well as TSH and ferritin level at the end of therapy) were entered. As this was an interventional study, the first model also contained the study arm as a possible influencing factor. In models 2–4, the study arm was neglected because it did not show a *p* < 0.05 in univariable regression analysis. Additionally, dosimetrically relevant parameters from previously published NTCP models or known influencing factors were included in models 1–3 (mean thyroid dose, baseline thyroid volume, V45 Gy, and V30 Gy) [6, 7, 28, 29]. The multivariable regression model was estimated in MedCalc by applying stepwise backward selection. Only parameters with a *p* < 0.05 remained within the model. All entered parameters from the multivariable logistic regression analysis were checked not to interact with each other by means of Spearman’s rank correlation coefficient.

We chose the area under the curve (AUC) from receiver operating characteristics (ROC) as a performance measurement of our prediction model [30]. An AUC of 0.8–1 is considered excellent, an AUC of 0.7–0.8 means good performance, less than 0.7 is considered suboptimal, and an AUC of 0.5 corresponds to an unusable model. Cutoff values (e.g., for phase angle) were derived from ROC by calculating a Youden index.

For DVH mapping of the thyroid gland, the software tool DVH Analytics by Cutright et al. was employed [31]. Kaplan–Meier method was used to calculate the cumulative incidence of hypothyroidism and the overall survival applying MedCalc.

## Results

### Patients

**Fig. 1:**
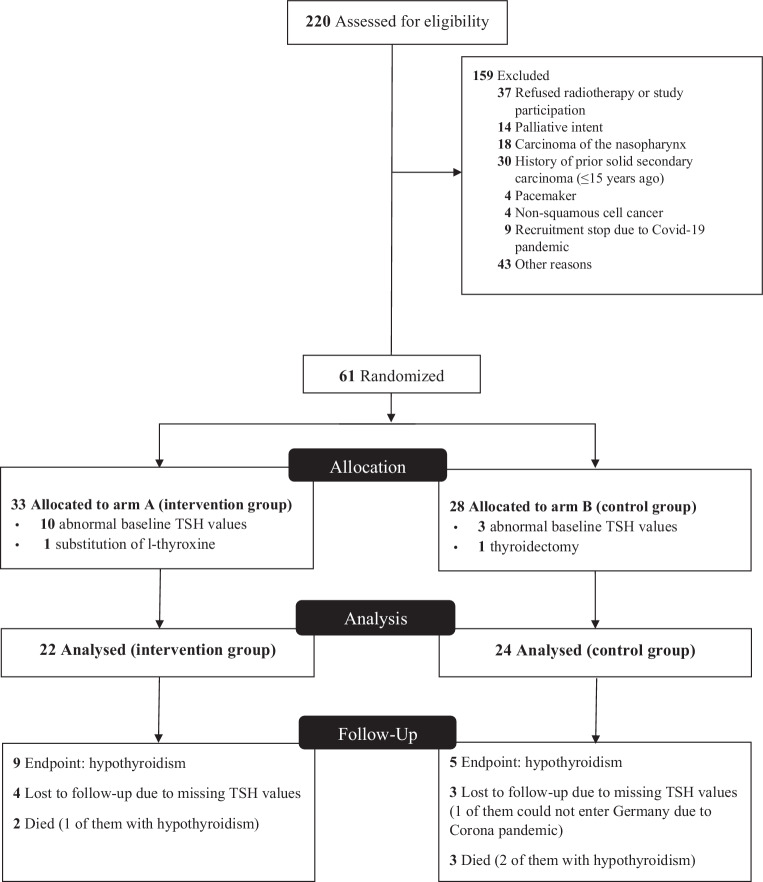
Consort diagram on patient selection [36]. Nutritional data and the initial screening of all 61 patients have been described previously elsewhere [20]

Between October 2018 and October 2020, 220 patients were screened for eligibility. 174 patients were considered unsuitable (see CONSORT diagram, Fig. 1). Clinical data from the remaining 46 patients were investigated.

**Fig. 2:**
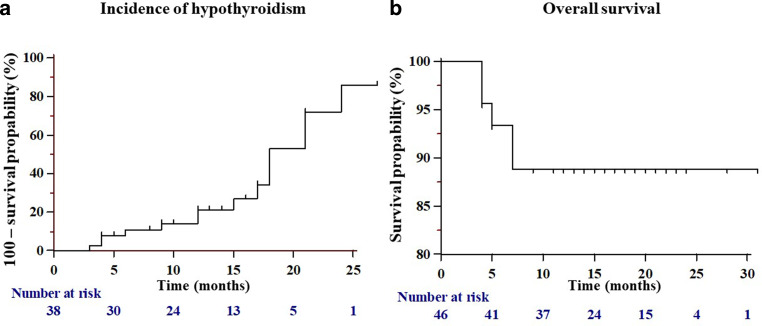
Kaplan–Meier curves on the cumulative incidence of hypothyroidism (**a**) and overall survival (**b**) for all patients. Cumulative 1‑ and 2‑year incidence for hypothyroidism was 21.3% and 85.9%, respectively. For overall survival, cumulative percentage of alive patients was 88.9% after 7 months

In all patients, median age was 63 [57; 73] years with a predominance of male patients of approximately 2:1. Baseline patient characteristics are shown in Table 1. Overall follow-up was 16.5 [12; 22] months and TSH-specific follow-up was 13.5 [8.8; 17] months (see Fig. 2).

**Table 1:** Pretreatment baseline patient characteristics

	Arm A Intervention group		Arm B Control group		
	Number	%	Number	%	*p*-value
	*n* = 22	47.8	*n* = 24	52.2	0.88
*Age*					0.96
≤ 60 years	9	40.9	10	41.7	
> 60 years	13	59.1	14	58.3	
*Gender: Male*	16	72.7	16	66.7	0.75
*Karnofsky performance status*					0.35
< 80%	1	4.5	4	16.7	
≥ 80%	21	95.5	20	83.3	
*Diabetes mellitus*	0	0	3	12.5	0.24
*Primary site*					0.07
a) Oropharynx	16	72.7	12	50	
b) Oral cavity	1	4.5	7	29.2	
c) Hypopharynx	0	0	3	12.5	
d) Larynx	3	13.6	2	8.3	
e) Other	2	9	0	0	
*UICC classification*					0.96
I	8	36.4	8	33.3	
II	4	18.2	6	25	
III	3	13.6	3	12.5	
IV	7	31.8	7	29.2	
*T‑classification*					0.15
T1	2	9.1	6	25	
T2	12	54.5	9	37.5	
T3	2	9.1	6	25	
T4	6	27.3	3	12.5	
*N‑classification*					0.3
N0	3	13.6	5	20.8	
N1	9	40.9	11	45.8	
N2	7	31.8	8	33.3	
N3	3	13.6	0	0	
**(Chemo)radiotherapy**					
*Treatment mode*					0.56
a) Primary					
– Concurrent chemotherapy	10	45.5	7	29.2	
– RT alone	2	9	3	12.5	
b) Adjuvant					
– Concurrent chemotherapy	6	27.3	5	20.8	
– RT alone	4	18.2	9	37.5	
*Concurrent chemotherapy (as initially prescribed):*					0.42
Cisplatin 100 mg/m^2^ 3‑weekly	4	18.2	5	20.8	
Cisplatin 40 mg/m^2^ weekly	9	40.9	7	29.2	
5‑FU/mitomycin C	2	9.1	0	0	
Cetuximab	1	4.5	0	0	
*Cisplatin dose*					0.67
< 200 mg/m^2^	3	13.6	4	16.8	
≥ 200 mg/m^2^	10	76.9	8	33.3	
*RT to the neck*					0.42
Unilateral	2	9	5	20.8	
Bilateral	20	90.9	18	75	
None	0	0	1	4.2	
*RT to neck level IV or VI*	20	90.9	22	91.7	1
**Nutritional factors**					
*∆ calorie deficit (kcal)*	117.8 [−661.5–737.1]	–	119.5 [−383.8; 660.1]	–	0.88
*BMI (kg/m*^*2*^*)*	24.1 ± 5.4	–	24.6 ± 4	–	0.52
*FFMI (kg/m*^*2*^*)*	18.2 [15.9; 19.3]	–	17.5 [16.2; 20.7]	–	0.75
*Phase angle (°)*	5.1 ± 1.2	–	5 ± 0.9	–	0.5
*Iodine intake (µg)*	175.5 [79.5; 204.5]	–	126 [94; 205.3]	–	0.9
*Protein intake (g)*	82.2 [60.8; 109.9]	–	84 [64.5; 106.2]	–	0.63
*Iron intake (mg)*	11 [8.9; 17.6]	–	12.8 [11.7; 17.4]	–	0.08
*Selenium intake (µg)*	12.6 [0; 57.3]	–	0 [0; 0]	–	0.023^*a*^
**Laboratory analyses**					
*TSH (reference: 0.55–4.78* *mU/L)*	1.49 [0.9–2.1]	–	1.21 [1; 1.80]	–	0.66
*Ferritin (reference: 10–291* *µg/L)*	130.4 [51.8; 418.3]	–	126.5 [66.8; 263.3]	–	0.8
*Hemoglobin (reference: 12.3–15.3* *g/dL)*	13.1 [12.4; 13.7]	–	12.7 [11.3; 14.2]	–	0.5
*Total protein (reference: 57–82* *g/L)*	71.8 [68.3; 77.2]	–	73.6 [69.2; 97.7]	–	0.37
**Volumetric and dosimetric features**					
*Volume of thyroid gland (cm*^*3*^*) in planning CT*	15.6 [10.6; 21.9]	–	14 [9.2; 20.5]	–	0.64
*Prescribed PTV high dose*					0.96
60 Gy	5	22.7	6	25	
66 Gy	6	27.3	7	29.2	
≥ 70 Gy	11	50	11	45.8	
*D*_*mean*_* of thyroid gland (Gy)*	50 ± 12.2	–	46.8 ± 13.9	–	0.52
*D*_*min*_* of thyroid gland (Gy)*	40.8 [22.1; 46.2]	–	37.3 [9.2; 48.3]	–	0.74
*D*_*max*_* of thyroid gland (Gy)*	62.5 [56.2; 68]	–	62.7 [57.1; 66.4]	–	0.81

*RT* radiotherapy,* UICC* Union Internationale Contre le Cancer, *5‑FU* 5-fluorouracil,* ∆ calorie deficit* difference between the patient’s actual calorie intake and the calculated calories required to maintain body weight (kcal),* BMI* body mass index,* FFMI* fat-free mass index,* TSH* thyroid-stimulating hormone,* PTV* planning target volume, *D*_*mean*_ *(Gy)* mean dose, *D*_*min*_* (Gy)* minimum dose,* D*_*max*_ maximum dose

^a^Regarding selenium intake at the end of therapy, no differences were present any more between the two groups (*p* = 0.92). TNM staging was performed according to UICC 8. Values in the square brackets correspond to the first and third quartiles of the interquartile range (IQR).

### Analysis of thyroid function

At baseline, all patients were euthyroid. Median baseline TSH was 1.3 [1; 2] mU/L and decreased to 0.9 [0.4; 1.9] mU/L (reference: 0.55–4.78 mU/L) until therapy end (*p* = 0.02). At the end of therapy, only 1 patient in the control group presented with an elevated TSH level (*p* = 1), while 6 patients in each therapy arm had suppressed TSH levels below 0.55 mU/L (*p* = 1). During follow-up, 3 of these 12 patients with suppressed TSH levels developed subclinical or manifest hypothyroidism, 5 became euthyroid, and in 4 cases no follow-up TSH value was obtainable. Overall, 9 patients of the intervention and 5 patients of the control group presented with elevated TSH values (without differentiating between manifest and subclinical forms) during follow-up (*p* = 0.202). These 14 patients presented with a median TSH of 7.04 [5.45; 8.23] mU/L. For hypothyroidism, cumulative percentage for the 1‑ and 2‑year incidence was 21.3% and 85.9%, respectively (see Fig. 2**)**.

### Analysis of nutritional status

For all patients (*n* = 46), median baseline BMI was 23 [21; 27.5] kg/m^2^ and dropped to 22.2 [20.8; 25.2] kg/m^2^ at the end of therapy (*p* < 0.001). No differences in BMI reduction were found between the treatment arms (*p* = 0.36). Baseline FFMI and phase angle were 18 [16.2; 20.1] kg/m^2^ and 5.2 [4.7; 6.1]°, respectively. At therapy completion, both measured values dropped to 17.4 [15.5; 17.4] kg/m^2^ and 5 [4.4; 5.9]° for median FFMI (*p* < 0.001) and phase angle (*p* = 0.2), respectively. The pre- versus posttherapeutic changes in FFMI and phase angle did not differ between the study arms (FFMI: *p* = 0.57; phase angle: *p* = 0.59). Until the end of therapy, the baseline median ∆ calorie deficit increased from 117.8 [−439; 708.7] kcal to −539.6 [−672.6; 109] kcal under ongoing (chemo)radiotherapy in the entire patient cohort (*p* = 0.02).

In all patients, no relevant differences between the median baseline iodine intake of 138 [88.5; 201.8] µg and 133 [80.5; 204] µg at therapy completion were observed (*p* = 0.92), while the baseline median oral protein intake decreased from 84 [62.3; 108.4] g to 70.6 [46.5; 87.4] g (*p* = 0.028). The overall iron intake was 11 [8.9; 17.6] mg at baseline and fell to 11.9 [7.9; 14.1] mg at the end of therapy (*p* = 0.8). Initially, overall oral selenium intake was measured to be 0 [0; 35.4] µg at baseline and 39.2 [0; 67] µg at the end of therapy (*p* = 0.002). Considering pre- and posttherapeutic changes (∆ x), we did not observe relevant differences between the study arms (∆ iodine intake: *p* = 0.76; ∆ protein intake: *p* = 0.36; ∆ iron intake: *p* = 0.8; ∆ selenium intake: *p* = 0.21).

In all patients, baseline ferritin was 126.9 [54; 277.5] µg/L and increased to 237 [86; 417] µg/L by the end of therapy (*p* = 0.001), without differences between the treatment arms (*p* = 0.11). Within the entire cohort, baseline hemoglobin was 12.8 [11.8; 14.0] g/dL and baseline total protein 72.2 [68.9; 87.3] g/L. Both parameters decreased, to 11.1 [9.8; 13.1] g/dL (for hemoglobin: *p* < 0.001) and to 68 [62.3; 71.5] g/L (total protein: *p* < 0.001).

### Volumetric and dosimetric evaluation

In all 46 planning CTs, median thyroid volume was 14.7 [9.9; 21] cm^3^. Additionally, 114 thyroid glands were contoured on pre- and posttherapeutic MRIs and CTs: An intramodal comparison by means of pre- and posttherapeutic MRI comparing thyroid volume was possible in 19 patients (41.3%), and pre- and posttherapeutic CT in 10 patients (21.7%). 17 patients (37%) were not included into this subanalysis, 5 (10.9%) of whom had died during follow-up. In the remaining 12 cases, either only an intermodal comparison would have been possible, or patients did not receive further radiological follow-up examinations. The median time interval between these pre- and posttherapeutic radiological examinations was 9 [5; 15] months. At baseline, the MRI-based comparison revealed a thyroid volume of 13.1 [10.9; 20.5] cm^3^, which then decreased to 10.8 [10.8; 17] cm^3^ during follow-up. The CT-based comparison revealed an initial thyroid volume of 15.5 [12; 22.7] cm^3^. The CT-based thyroid volume during follow-up was 12.5 [12.5; 16.2] cm^3^. In the entire cohort, a relevant volume loss occurred during follow-up (*p* = 0.004) without differences between the study arms (*p* = 0.62).

**Fig. 3:**
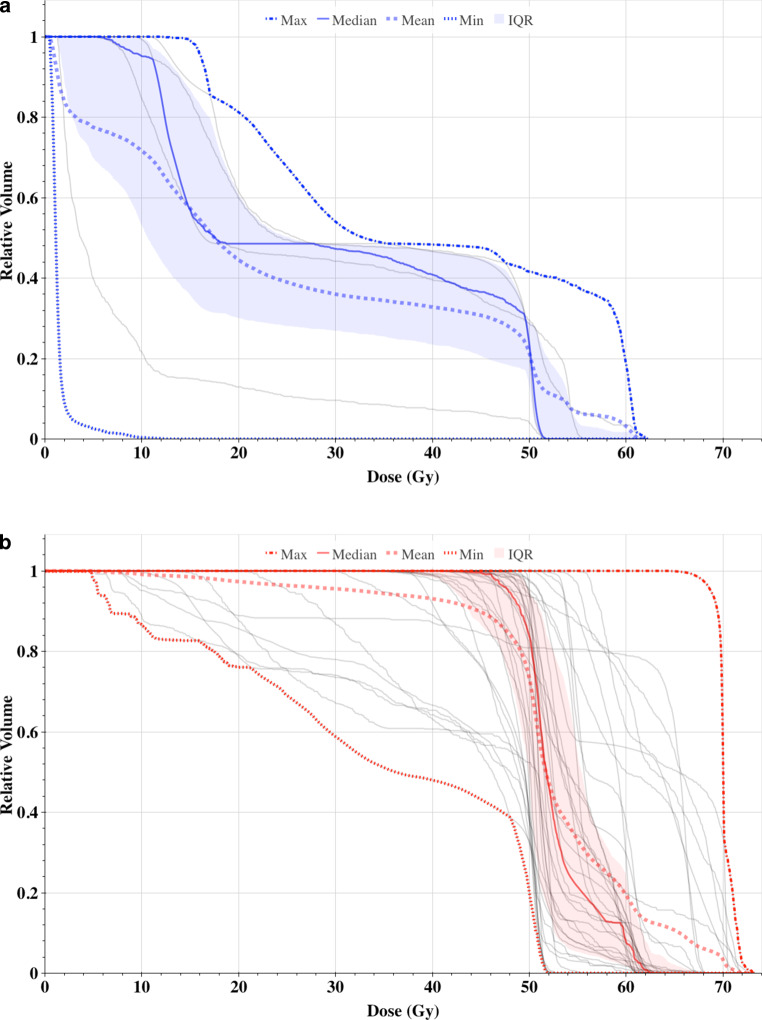
Dose–volume histograms for the thyroid gland in unilaterally (**a**, *blue*) and bilaterally (**b**, *red*) irradiated patients. *IQR* Interquartile range

Table 1 summarizes the main dosimetric parameters and Fig. 3 shows the comparison of thyroid DVH for the entire patient cohort.

### Uni- and multivariable analysis: normal tissue complication probability (NTCP) model

Before starting the univariable regression analysis, previously published models were tested on our dataset. Firstly, we tested the model by Rønjom et al. [28], receiving an AUC of 0.77 (95% CI: 0.62–0.88) with a sensitivity of 85.7% and specificity of 62.5% (Youden index: 0.48; *p* = 0.001 for area = 0.5). Secondly, we applied the proposed NTCP model by Cella et al., who had published two different models. Our chosen model contained gender and V30 Gy (cm^3^) as candidate variables. We received an AUC of 0.76 (95% CI: 0.61–0.87), and a sensitivity of 78.6% and specificity of 71.9% (Youden index: 0.5; *p* = 0.001 for area = 0.5). Thirdly, the NTCP model by Boomsma et al. was tested on our dataset to give an AUC of 0.78 (95% CI: 0.63–0.89) and, taking a Youden index of 0.5, a sensitivity of 78.6% and specificity of 71.9% resulted (*p* < 0.001 for area = 0.5).

Results from the univariable regression analyses with all variables are shown in Table 2. We did not identify any dosimetric factors associated with the development of hypothyroidism. The following variables showed *p*-values < 0.05: baseline iodine intake, ∆ iodine intake, baseline TSH and baseline ferritin level, as well as TSH and ferritin levels at the end of therapy. These parameters were entered into four consecutive multivariable regression models.

**Table 2:** Results from the univariable and multivariable logistic regression analysis

	Univariable logistic regression analysis			
	Regression coefficient	Odds ratio	95% CI of the odds ratio	*p*-value
**General characteristics**				
*Treatment arm*				0.15
Intervention group	−0.97	0.38	0.1–1.4	
Control group	N/A	0.38	N/A	
*Gender*				0.61
Male	−0.35	0.7	0.19–1.68	
Female	N/A	1.42	N/A	
*Age*				0.43
≤ 60 years	0.51	1.77	0.47–5.93	
> 60 years	N/A	0.6	N/A	
*Karnofsky performance status (continuous)*	0.17	1.0	0.96–1.1	0.59
*T‑classification*				0.58
T1–2	−3.6	0.7	0.19–2.53	
T3–4	N/A	1.43	N/A	
*N‑classification*				0.71
N0	N/A	0.72	N/A	
N1–3	0.33	1.39	0.24–7.89	
*Setting*				0.28
Definitive	−0.71	0.49	0.13–1.79	
Adjuvant	N/A	2.04	N/A	
*Concurrent systemic treatment*				0.73
Radiotherapy only	N/A	1.25	N/A	
Chemo-/immunoradiotherapy	−0.22	0.8	0.22–2.87	
*Cumulative cisplatin dose*				0.96
< 200 mg/m^2^	N/A	1.04	N/A	
≥ 200 mg/m^2^	−0.04	0.96	0.14–6.67	
*Diabetes mellitus*^a^				0.99
Yes	−20.48^a^	–^a^	0–0.07^a^	
No	N/A	–	N/A	
*RT to neck level IV or VI*				0.81
Yes	N/A	1.35	N/A	
No	−0.3	0.74	0.07–7.84	
*RT to the neck*^a^				1
Unilateral	−20.7^a^	–^a^	0–0.07^a^	
Bilateral	N/A	–	N/A	
**Nutritional factors**				
*∆ calorie deficit (kcal) at two different time points* *(continuous variable)*				
Baseline	0	1	1–1.001	0.25
End of therapy	0	1	1–1.001	0.72
**Iodine intake (µg) at two different timepoints** **(continuous variable)**				
**Baseline**	**0.01**	**1.01**	**1–1.01**	**0.047**
End of therapy	−0.003	1	0.99–1	0.4
**∆ iodine intake**	**0.005**	**1.01**	**1.1.01**	**0.042**
*Protein intake (g) at two different timepoints* *(continuous variable)*				
Baseline	0.01	1.01	0.99–1.02	0.35
End of therapy	−0.01	0.99	0.98–1.01	0.51
*Iron intake (mg) at two different timepoints* *(continuous variable)*				
Baseline	0.05	1.05	0.92–1.19	0.49
End of therapy	−0.06	0.23	0.86–1.04	0.23
*Selenium intake (µg) at two different timepoints* *(continuous variable)*				
Baseline	0.01	1.01	0.98–1.03	0.68
End of therapy	0	1	0.98–1.02	0.96
*BMI (kg/m*^*2*^*) at four different timepoints* *(continuous variable)*				
Baseline	0.07	1.07	0.94–1.22	0.31
End of therapy	0.07	1.08	0.92–1.26	0.36
First follow-up (6–8 weeks after therapy)	−0.21	0.81	0.6–1.09	0.17
Second follow-up (8 months after therapy)	0.002	1	0.74–1.35	0.99
*FFMI (kg/m*^*2*^*) at four different timepoints* *(continuous variable)*				
Baseline	−0.01	0.99	0.78–1.25	0.92
End of therapy	−0.04	0.96	0.71–1.29	0.79
First follow-up (6–8 weeks after therapy)	−0.21	0.81	0.6–1.09	0.17
Second follow-up (8 months after therapy)	−0.24	0.79	0.53–1.19	0.26
*Phase angle (°) at four different timepoints* *(continuous variable)*				
Baseline	−0.11	0.9	0.48–1.69	0.73
End of therapy	−0.17	0.85	0.46–1.55	0.59
First follow-up (6–8 weeks after therapy)	−0.81	0.44	0.19–1.06	0.07
Second follow-up (8 months after therapy)	−0.71	0.49	0.17–1.39	0.18
**Laboratory analyses**				
**TSH (mU/L)**				
**– Baseline**	**1.34**	**3.82**	**1.32–11.02**	**0.013**
**– End of therapy**	**0.74**	**2.09**	**1.1–3.98**	**0.024**
**–** ∆ TSH	−0.47	0.46	0.34–1.15	0.13
**Ferritin level (µg/L)**				
**– Baseline**	**0.004**	**1.004**	**1–1.008**	**0.048**
**– End of therapy**	**0.003**	**1.003**	**1–1.006**	**0.042**
**–** ∆ ferritin	0.002	1	1–1.01	0.39
*Total protein (g/L)*				
Baseline	0.07	1.07	0.97–1.19	0.19
End of therapy	0.06	1.06	0.96–1.17	0.26
*Hemoglobin (g/dL)*				
Baseline	−0.34	0.72	0.46–1.11	0.14
End of therapy	−0.22	0.8	0.59–1.11	0.18
**Volumetric and dosimetric indicators of the thyroid gland (at baseline)**				
Volume (cm^3^) in planning CT (continuous)	−0.1	0.9	0.81–1.01	0.07
***Dosimetric indicators (Gy; continuous)***				
Prescribed PTV high dose	−0.08	0.93	0.79–1.09	0.35
D_mean_	0.04	1.04	0.98–1.11	0.2
D_min_	0.04	1.04	1–1.09	0.051
D_max_	0.01	1.01	0.94–1.09	0.73
D99%	0.04	1.04	1–1.08	0.08
D98%	0.04	1.04	1–1.08	0.09
D50%	0.04	1.04	0.98–1.1	0.21
D95%	0.02	1.02	0.93‑1.12	0.64
D1%	0.02	1.02	0.95‑1.1	0.61
D2%	0.02	1.02	0.95–1.1	0.63
***VxGy (cm***^***3***^***; continuous)***				
V10Gy	−0.06	0.94	0.86–1.03	0.16
V20Gy	−0.05	0.95	0.88–1.03	0.25
V30Gy	−0.04	0.96	0.89–1.04	0.29
V40Gy	−0.04	0.96	0.89–1.04	0.33
V45Gy	−0.03	0.97	0.9–1.05	0.4
V50Gy	−0.02	0.98	0.9–1.07	0.67
V60Gy	−0.05	0.95	0.8–1.13	0.56
V70Gy	0.003	1	0.53–1.9	0.99
***CVxGy (cm***^***3***^***; continuous)***				
CV10Gy	−0.62	0.54	1.3–2.27	0.4
CV20Gy	−0.33	0.72	0.44–1.19	0.19
CV30Gy	−0.25	0.78	0.55–1.1	0.16
CV40Gy	−0.22	0.8	0.59–1.08	0.14
	**Multivariable regression analysis**			
	**Regression coefficient**	**Odds ratio**	**95% CI of the odds ratio**	***p*-value**
**Model 1**				
Baseline ferritin (µg/L)	0.004	1.004	1–1.01	**0.04**
Constant	−1.62	N/A	N/A	N/A
**Model 2**				
Baseline ferritin (µg/L)	0.004	1.004	1–1.01	**0.04**
**Model 3**				
Baseline ferritin (µg/L)	0.005	1.005	1–1.01	**0.05**
Baseline TSH (mU/L)	1.26	3.5284	1.2–10.41	**0.02**
Constant	−3.76	N/A	N/A	N/A
**Model 4**				
Baseline TSH (mU/L)	1.26	3.53	1.2–10.41	**0.02**
Baseline ferritin (µg/L)	0.01	1.01	1–1–01	**0.05**
Constant	−3.76	N/A	N/A	N/A

Results from the univariable and multivariable logistic regression analysis for hypothyroidism. Parameters with *p* < 0.05 are written in **bold**

*N/A* not applicable, *RT* radiotherapy, *TSH* thyroid-stimulating hormone, *95% CI 95%* confidence interval, *BMI* body mass index, *FFMI* fat-free mass index, *VxGy* thyroid volume (cm^3^) receiving x Gy, *CVxGy (cm*^*3*^*)* thyroid volume receiving ≤ x Gy

^a^None of the diabetic and none of the unilaterally irradiated patients presented with the endpoint hypothyroidism. Therefore, the 95% CI was calculated applying the “rule of three” [35]

Model 1 tested all variables from our univariable regression analysis with *p* < 0.05, together with D_mean_, thyroid volume V45 Gy, V30 Gy, and the study arm, showing an AUC of 0.68 (0.52–0.82). Model 2 consisted of all univariable parameters with *p* < 0.05, D_mean_, thyroid volume, V30 Gy, and V45 Gy. This model revealed an AUC of 0.68 (95% CI: 0.52–0.82). Model 3 included our univariable parameters (with *p* < 0.05), D_mean_, and thyroid volume. This model showed an AUC of 0.78 (95% CI: 0.63–0.89). Model 4 consisted of all univariable parameters with *p* < 0.05. Here, ROC analysis revealed an AUC of 0.78 (95% CI: 0.63–0.89). Results from the univariable and multivariable regression analyses are summarized in Table 2.

Our final NTCP model is built on two parameters, namely baseline TSH and ferritin level, as they showed the best predictive potential. Prior to running our multivariable analyses, correlation between these parameters was excluded (correlation coefficient r = 0.07; *p* = 0.64). We derived the following NTCP model:

$$NTCP=\left(1+e^{-S}\right)^{-1},\text{in which}$$

$$S=-3.76+\left(1.26\cdot \textit{baseline}\,TSH\right)+(0.01\cdot \textit{baseline}\,\textit{ferritin}\,\textit{level})$$

**Fig. 4:**
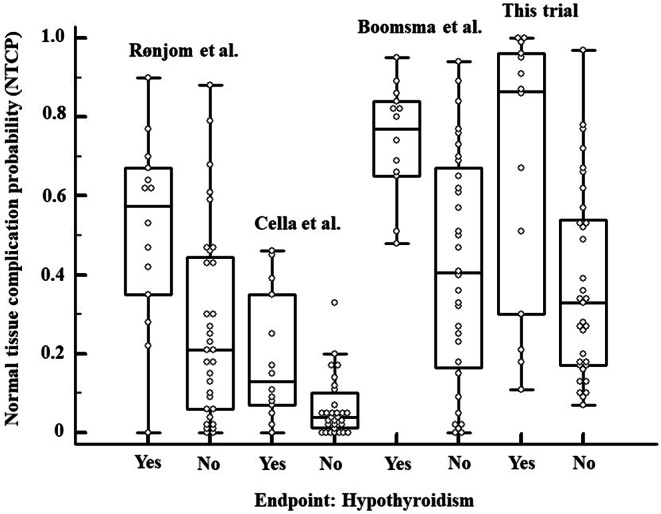
Boxplot diagrams on the prediction of hypothyroidism applying different NTCP models from Rønjom [28], Cella [29] (only one of the two published models was tested), and Boomsma et al. [7]. Every dot marks one case

Application of this NTCP model to our patient population showed an AUC of 0.76 (95% CI: 0.61–0.87), with a sensitivity of 57.1% and specificity of 96.9% calculated for a Youden index of 0.73 (*p* = 0.004; area = 0.5). The performance of the present NTCP model compared to other previously published NTCP models can be seen in Fig. 4.

## Discussion

This is the first prospective study including nutritional parameters to develop a new multivariable NTCP model for radiation-induced hypothyroidism. In all previous publications, dosimetric factors always played a decisive role. According to Boomsma and Rønjom et al., D_mean_ and the initial thyroid volume were predictors for hypothyroidism [7, 28]. In accordance, Emami et al. assumed the onset of hypothyroidism when 2/3 of the thyroid gland was damaged [4]. Although we noticed a reduction in thyroid volume during follow-up, we could not prove thyroid volume to be an independent predictor of hypothyroidism. Still, both models showed good performance in our dataset (Boomsma: AUC = 0.78, Rønjom: AUC = 0.77).

To allow a dosimetric approach, we included dosimetric and volumetric parameters in models 1–3, analogous to previously published data (D_mean_, thyroid volume, V30 Gy, and V45 Gy) [2, 5–8, 28, 29]. These parameters were eliminated from all three models and only baseline TSH and ferritin level prevailed (see Table 2). Although TSH was the leading factor in our NTCP model (multiplication factor for TSH was 1.26 vs. 0.01 for ferritin), both increased TSH and ferritin levels were associated with an increasing risk of hypothyroidism. Although Rønjom et al. also tested the influence of baseline TSH, it was a nonpredictor for hypothyroidism [28]. Overall, many of our investigated parameters have simply not been tested before [7, 28, 29], and the influence of these parameters remains unclear, suggesting an underrepresentation in previous NTCP models.

The undersupply of certain macro- and micronutrients appears to be a crucial risk factor for thyroid dysfunction [10–13, 15, 32]. Iron deficiency is one of the most common deficiency symptoms worldwide, which may manifest not only as iron deficiency anemia, but also in the form of low ferritin levels, thus negatively affecting thyroid metabolism [12, 13, 32]. This mainly happens through reduced thyroid hormone synthesis by the heme-dependent enzyme thyroid peroxidase (TPO) [11, 12, 32]. Conversely, in our patients, higher baseline ferritin positively correlated with hypothyroidism. Ferritin is also known as acute-phase protein. It isolates iron, protecting it from bacterial abduction to stop further bacterial growth. During inflammatory or malignant processes, ferritin is upregulated to hide free iron from cancer cells or bacteria [33]. Since all our patients suffered from an underlying malignant disease, higher ferritin levels were common. If we now relate the iron and ferritin balance, we suppose that higher ferritin levels lead to lower iron levels in the long term, and thus interfere with thyroid hormone synthesis by influencing heme-dependent TPO. Iodine intake is considered another crucial factor for thyroid metabolism because it constitutes 59% and 65% of fT3 and fT4, respectively [11]. Although we expected a certain influence of iodine intake on the development of hypothyroidism, we could not prove this in our multivariable analysis.

As the risk for radiation-induced hypothyroidism increases with time, Boomsma et al. reported on a cumulative 2‑year incidence of 36%. In comparison, we observed a cumulative 1‑ and 2‑year incidence of 21.3% and 85.9%, respectively. We are aware that especially our 2‑year incidence is noticeably higher than that of Boomsma et al. We believe that this is partly biased by our shorter TSH-specific follow-up time of 13.5 [8.8; 17] months (Boomsma et al.: 2.49 years, range: 0.33–3.30 [7]) and by the underdiagnosis of hypothyroidism during the corona pandemic. Due to the corona pandemic and during the general lockdown in XXX, blood sampling for TSH follow-up took place later. Therefore, we suspect that this resulted in a steep increase in incidence after approximately 15 months. Our assumption is that hypothyroidism had been diagnosed earlier under normal conditions. Cella et al. retrospectively investigated the outcome of 53 patients with Hodgkin’s lymphoma. These authors reported on 41.5% of patients presenting with hypothyroidism after a median follow-up time of 32 months (range 6–99) after completion of radiotherapy [29]. For patients with nasopharyngeal cancer, Luo et al. reported on 22.4% of patients suffering from hypothyroidism after a median follow-up of 24 (range 3 to 66) months [34].

Overall, we proved a worsening of nutritional status in the pre- vs. posttherapeutic comparison. Macro- and micronutrient intake did not change during ongoing therapy, except for selenium and protein. As expected, protein intake decreased at the end of therapy (*p* = 0.028), while selenium intake increased (*p* = 0.002). The increased selenium intake might be related to the higher proportion of nutritional supplementation at the end of therapy, e.g., by sip feeds, as they are usually enriched with selenium.

The major limitation of the present study is its small patient number. Usually one would assume a dose–response relationship within the framework of the biological model. We expected a dose dependence in the occurrence of hypothyroidism after irradiation. The observation that none of the 7 patients with unilateral irradiation (implicitly lower dose) developed hypothyroidism suggests a dose effect. Since the group is small, with 7 patients, masking of the dose is conceivable. Nevertheless, no statistic differences were obtained in the comparison to bilaterally irradiated patients. Of course, it is important to critically question whether a dose dependence would be statistically present in a bigger patient cohort. To achieve a homogeneous patient population, we subjected our patients to a very strict selection process (see CONSORT diagram) and excluded all patients with possible pre-existing diseases of the thyroid gland from the analysis. In the statistical evaluation and calculation of a multivariable logistic regression model, all variables introduced into the model are crucial. Since there are various possible metabolic and nutritional factors influencing thyroid gland metabolism, we included them in our analysis. Some of these metabolic parameters were entered into a NTCP model for the first time. This could have led to an overestimation of the dose effect in contrast to previously undetected metabolic phenomena in earlier published data.

We conclude that laboratory chemical parameters, namely baseline TSH and ferritin, act as independent predictors for the occurrence of radiotherapy-associated hypothyroidism. The addition of laboratory chemical analyses to dosimetric and clinical parameters could improve the accuracy and validity of future NTCP models.
