# Correction to: The impact of nutritional counseling on thyroid disorders in head and neck cancer patients after (chemo)radiotherapy: results from a prospective interventional trial

**DOI:** 10.1007/s00066-021-01888-w

**Published:** 2021-12-09

**Authors:** Anastassia Löser, Kerstin Ramke, Maximilian Grohmann, Linda Krause, Pia Roser, Franziska Greinert, Anna Finger, Margaret Sommer, Eva Culmann, Tessa Lorenz, Saskia Becker, Marvin Henze, Daniel Schodrok, Julia von Grundherr, Silke Tribius, Andreas Krüll, Cordula Petersen

**Affiliations:** 1 Outpatient Center of the UKE GmbH, Department of Radiotherapy and Radiation Oncology, University Medical Center Hamburg-Eppendorf, Martinistraße 52, 20246 Hamburg, Germany; 2 Institute of Medical Biometry and Epidemiology, University Medical Center Hamburg-Eppendorf, Martinistraße 52, 20246 Hamburg, Germany; 3 Center for Internal Medicine, Department of Nephrology, Rheumatology and Endocrinology, University Medical Center Hamburg-Eppendorf, Martinistraße 52, 20246 Hamburg, Germany; 4 University Medical Center Hamburg-Eppendorf, University Cancer Center Hamburg (UCCH), Martinistraße 52, 20246 Hamburg, Germany; 5 Hermann Holthusen Institute for Radiation Oncology, Asklepios Hospital St. Georg, Lohmühlenstraße 5, 20099 Hamburg, Germany; 6 Department of Radiotherapy and Radiation Oncology, University Medical Center Hamburg-Eppendorf, Martinistraße 52, 20246 Hamburg, Germany

**Correction to:**

**Strahlenther Onkol 2021**

10.1007/s00066-021-01865-3

The original version of this article unfortunately contained a mistake.

There is a mistake in the legend of Fig. 3. The labelling has been reversed. It should be: Fig. 3 Dose-volume histograms for the thyroid gland in unilaterally (**a** *BLUE*) and bilaterally (**b** *RED*) irradiated patients. Instead of: Fig. 3 Dose–volume histograms for the thyroid gland in unilaterally (**a** *red*) and bilaterally (**b** *blue*) irradiated patients.

The original article has been corrected.

